# Analysing Educational Interventions with Gifted Students. Systematic Review

**DOI:** 10.3390/children8050365

**Published:** 2021-05-03

**Authors:** Inmaculada García-Martínez, Rafaela Gutiérrez Cáceres, Antonio Luque de la Rosa, Samuel P. León

**Affiliations:** 1 Department of Didactics and School Organization, University of Granada, Campus de Cartuja s/n, 18071 Granada, Spain; 2 Department of Education, University of Almería, Carratera Sacramento s/n, 04160 Almería, Spain; rcaceres@ual.es; 3 Department of Pedagogy, University of Jaén, Campus Las Lagunillas s/n, 23071 Jaén, Spain; sparra@ujaen.es

**Keywords:** educational intervention, gifted students, learning problems, behavioral problems, systematic review

## Abstract

(1) Background: Educational attention to gifted students has not been a well-established line of research due to the multiple conceptions about their characterisation. While educational attention has tended to respond to students who present learning difficulties due to their limitations, it has been observed that gifted students may also fail in their studies. The purpose of this study is to examine educational interventions carried out with this population worldwide; (2) Methods: The methodological design is a systematic review, following the PRISMA guidelines, in the Scopus and WOS databases on educational interventions and gifted students; (3) Results: The papers were studied through a qualitative content analysis based on a population of 557 articles, with a final sample of 14, finding a great variety of didactic strategies and models oriented to meet the needs of this group. In relation to the quality of the studies, the lack of pre-post methodological designs focused on performance stands out; (4) Conclusions: Educational research with gifted population demands more interventions personalised to the specific characteristics of the students. In addition, there is a need for further research with quasi-experimental designs with this population to identify quality, not generalised, interventions to meet these needs and replace them with individualised adaptations regarding the needs and interests of these students in order to increase their motivation and reduce failure.

## 1. Introduction

Attention to diversity is nowadays one of the challenges to be faced by any education professional. Traditionally, the concept “attention to diversity” has merely focused on people with disabilities [1]. However, throughout the years, attention to diversity has been extended to other groups of students with specific educational support needs, including those with high intellectual abilities. In this way, education systems in the 21st century are attempting to provide a successful educational response to those who need a “readjustment” of the teaching performance. This would undoubtedly enable all people to reach their full potential. Within this group of students with specific educational support needs, the group of people with high abilities or giftedness have been relegated to not being seen as a priority. This response may sometimes be inexistent. Furthermore, it frequently seems to be inadequately adapted to the learning needs of these students [2]. Such intervention is also necessary to ensure in all cases the best possible development of each student’s abilities.

Different research approaches have thus focused on the study of giftedness in recent years. Nevertheless, no widely accepted definition for this concept has been found [3]. Generally speaking, pupils who have high cognitive abilities to achieve high performance in school are classified as high ability. This association corresponds to the traditional approach to giftedness and it is based on cognitive ability as the sole factor. In contrast, this association has now been broadened to be considered as a multidimensional construct which includes several characteristics of a person, such as high general cognitive ability, academic achievement, creativity or motivation. This finding has throughout time led to a shift from a traditional approach to one which considers other factors. As a consequence, different characteristic terms within this group now present a tendency to coexist. More specifically, these terms are High Ability, Gifted, Talented, Highly Able, Specially Able, Gifted, or Highly Capable [2]. One of the most widely accepted definitions was the one established in the Marland Report, referring to students with a high level of performance in any of the following abilities or aptitudes, alone or in combination: (1) intellectual ability, (2) specific academic aptitude, (3) creative or productive thinking, (4) leadership ability, (5) visual aptitude and performance in art, and (6) psycho-motor ability [4]. But certainly the most generally accepted definition has been provided by Renzulli [5,6], who considers gifted student to be those who possess three sets of characteristics with an equal emphasis on each of them: above-average intellectual ability; a high level of dedication to tasks; high levels of creativity.

On the other hand, there are different models for analysing giftedness and the identification of its diagnostic factors [3,7,8]. However, there are common characteristics in all of them which have never been considered, primarily those associated with intellectual competence [9]. Thus, a great variety of explanatory models focus on the cognitive component as a determining factor for diagnosis [3,10,11,12]. Other models focus on the socio-cultural component, i.e., the family and social context in which the individual develops [13,14,15], among other models.

Despite the discrepancies between the approaches of these theoretical models, they all agree in understanding giftedness as a multidimensional construct. Concerning this, it is important to focus on the diversity of areas (cognitive, social and emotional) in order to make an adequate diagnosis and establish measures for action [2,5]. In addition, according to Tourón et al. [16], the crucial point in the case of this type of student is not to determine a precise diagnosis of giftedness and all its components—only from an interdisciplinary approach is it possible to promote the student’s full development, but to have the necessary human and material resources available at an educational level to provide an appropriate response which promotes both their maximum academic and social development. Given the existence of an associated neurological and socioemotional basis, this may have an impact on the self-esteem and motivation of these students as well as on their self-perception, academic performance and social integration [2,6,7].

In this regard, the developmental component plays a very important role in the analysis of these students’ abilities, as the school environment and the learning process of these students tend to influence the over-performance of cognitive skills. Likewise, environmental stimulation is another factor which has a considerable impact on the achievement of these students. In any case, high ability is not equivalent to good performance. In this regard, Barbier, Donche and Verschueren [9] developed a study in which they examined the inhibitors and facilitators associated with achievement in the Achievement Orientation Model (AOM) within the teaching and learning processes with high and low- achievement students in the transition from primary to secondary school.

In this context, it is necessary to address the needs of these students by providing an appropriate educational response. According to Barrera et al. [17], regular educational schools are the most suitable place to instruct this type of student, to avoid segregation or parallel systems, applying strategies which greatly promote the abilities of people with high abilities by adapting those used for each student [18,19,20,21,22].

According to Crisol et al. [23], these strategies can focus on aspects such as:

- The quantity of learning and teaching tasks.
- The most demanding expectations in terms of quality.
- The teaching style and its guiding and leadership role.
- Promotion of cooperation and responsibility.
- Promotion of social values and behaviour.

As a result, nowadays there is a tendency for different educational administrations to promote the improvement of educational attention in this group of students [3], thus enacting laws whose main aim is to establish different measures and strategies for attention to diversity according to specific needs. However, it is a reality that in educational schools and concerning teachers, there is a lack of training and resources required to promote the design and development of measures and strategies for educational support. However, curricular adaptation methods such as flexibility of the compulsory schooling period, individualised or small-groups educational attention during the school day when required or the development of enrichment programmes and curricular adaptations are usually promoted to this type of students [24,25,26].

The systematic review of Bailey et al. [27] on interventions aimed at improving the performance of gifted and talented students reported the tendency to use extracurricular measures with gifted and talented students, while arguing for the importance of combined measures. These students then receive a response tailored to their needs. Similarly, a recent meta-analysis [28] on the effect of low-achievement interventions in gifted students found that such programmes do not significantly improve the performance of gifted students, although they are more effective compared to the general population. In view of the above and given the importance of providing information and training to the teachers involved in order to develop a more personalised education for high-ability students, the present research aims to find out about the educational interventions carried out worldwide with gifted students, trying to delve into the curricular adaptations which are carried out and the students’ perception of these adjustments. In this regard, the research questions this review study aims to answer are the following:

- What kind of educational interventions are being developed with con high abilities/gifted students?
- What perceptions do high abilities/gifted students have about educational measures adopted by schools?
- What degree of methodological quality shows the evidence on intervention research for high abilities/gifted students?

## 2. Materials and Methods

The selected method was a systematic review in the Scopus and WOS databases, in which data were examined in order to obtain a comprehensive overview of the available information regarding a concrete issue. These databases were chosen because of its prestige and worldwide recognition, as well as the fact that they contain a large part of the research within the field of education [29]. After collecting some relevant papers, researchers analyzed them and compared the evidence these provided with similar literature [30].

An analysis of the studies finally included was conducted from a twofold perspective. On the one hand, descriptive information about the studies and their findings (author, objective, design, participants and intervention was extracted; on the other hand, the quality of the included studies using an adaptation of the quality scale developed by Ferrero et al. [31] was analyzed, for application in interventions based on Project-Based Learning [32]. This scale includes items which analyze methodological aspects related to the quality of educational interventions such as randomisation, experimental control or the measurement of the validity and reliability for the variables used. In the scale, each item may take three values: positive (the quality criterion is met), negative (the quality criterion is not met) and unknown (no available information, on the criterion).

### 2.1. Search Procedures

The present systematic review follows the PRISMA recommendations [33] (for further details of the PRISMA process see Supplementary Table S1). This research was conducted in the Scopus and WOS databases. The first author conducted a combined search of the Web of Science, and Scopus databases on 13 April 2021 conducted an electronic search entering the terms (educational and intervention) and (high and capacities) or (high and abilities) or (gift) or (exceptional and ability) or (exceptionally and able) and (students or children) into the topic field. The initial search result was limited to (a) only articles (b) written in English, (c) in the time period 2011–2021 and (d) with categories restricted to “education/educational research”. At this initial stage, the search yielded 691 articles. After removing duplicates, the initial search yielded 557 articles. The search is not registered.

**Figure 1:**
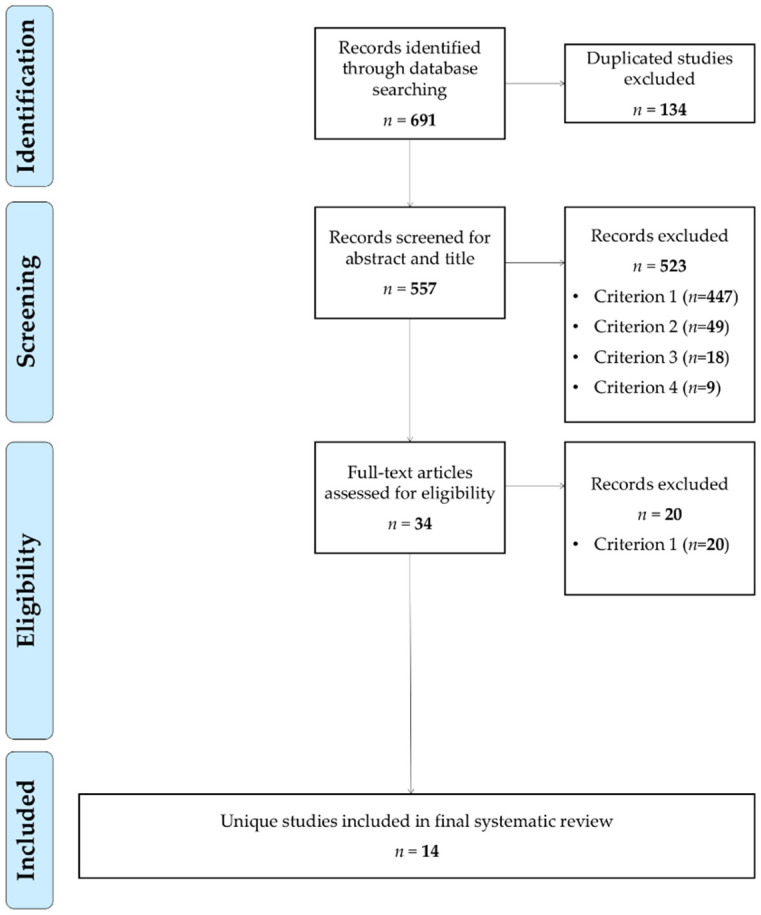
PRISMA flowchart.

In a first screening stage, the first author analysed the title and abstract of the 557 papers resulting from the first search by applying the inclusion criteria c1–c4 explained below. This first screening yielded 34 papers included for the next stage. The first and third authors independently did the full reading of these 34 papers to verify their inclusion according to the established criteria. The initial inter-rater agreement when cross-checking the results of both authors after the full reading was 98.31%. Disagreements were resolved by discussion and consensus between the two researchers until 100% agreement was reached. As a result of this phase, 14 papers were finally included in this systematic review (Table 1). Figure 1 represents the literature search process with a PRISMA flowchart.

Firstly, it was agreed upon to focus only on articles, as it was thought that these scientific papers would contain more synthetic and detailed research. It was also considered to include only articles in English, as this is the most widely scientifically accepted language. Similarly, it was considered appropriate to reduce the time range to the last 10 years to identify educational interventions with highly able/gifted students being current and relevant. Furthermore, given the fact that the focus was on education, the main aim was to identify educational interventions within the set of interventions performed on these groups of students. As a consequence it was considered necessary to restrict the search to the areas of Educational Research.

### 2.2. Selection Criteria

In order to identify educational interventions which were being performed with high capacity/high abilities/gifted students and to know how these perceived those implemented at their schools, it was necessary to establish some inclusion criteria to identify the studies. These criteria were (c1) Empirical studies which develop an educational intervention and whose objective sample were high capacity/high abilities or gifted students; (c2) at any educational stage; (c3) peer review articles and (c4) articles written in English. Criteria 1 was crucial, as its aim was to identify studies in which educational interventions were described and whose sample were high-capacity/high abilities or gifted students. On the other hand, studies whose samples were based on teachers, other professionals or parents were discarded. In order to obtain a general overview on the kind of measures and interventions to be performed by educational institutions, it was agreed upon to include some results from research carried out at any educational level: infant education, primary education, secondary education and university. Scientific rigour was added to this study by including only peer review articles, thus excluding books, book chapters, communications and thesis. At the same time, the search for reviews was focused on works whose full text was written in English, as it is the most used language for scientific purposes.

## 3. Results

### 3.1. Identification of the Selected Publications

The systematic review found 14 articles about interventions with gifted students or people with high abilities/high capacities/exceptionally able/exceptional ability. The main highlights of the manuscripts were extracted in Table 1, noting (1) author/s; (2) year of publication; (3) purpose; (4) participants; and (5) intervention.

### 3.2. Description of the Articles Included

The 14 studies included in the systematic review are very diverse. First, the research conducted by Dare, et al. [34] focuses on the perceptions and beliefs of high-ability students and students enrolled in inclusive classrooms about acceleration in inclusive school settings, through a group concept mapping. To do so, students had to rate the ideas generated by researchers about acceleration. Results revealed that there is some concern about the risk of exclusion of accelerated learners, despite the fact that this strategy is adapted to the educational needs of highly capable students. Similar findings were previously shown in another study by Dare and Nowicki [35], which collected the experience of students who had experienced acceleration in inclusive schools, providing their perceptions and attitudes towards this measure. Results indicated that the majority were in favour of this measure, since it suited their cognitive level, challenged them and prevented them from becoming unmotivated. Despite this positive assessment, the importance of the consolidation of an inclusive social climate, in which they are provided with opportunities and support for this adaptation, was indicated. Similarly, research performed by Dare, et al. [36] also included the students’ opinion and own experience with 26 high-ability students participating in the study, of whom 19 had accelerated. The results showed strong agreement with this measure, as it provided a better learning environment for those progressing from one year to the next when challenging activities depending on their abilities were performed. The emotional state of the student was identified as another important issue to consider prior to this measure, noting that it is essential that the student is motivated to change course in order to maintain his or her socio-emotional well-being. Another issue to be examined is the student’s proficiency in different subjects, noting that it is possible that he/she may not be outstanding in all subjects, but in any case, acceleration should be done in all subjects, with a view to challenging his/her cognitive ability. Peer group, context and support and social conditions were pointed out as other factors to consider before initiating this measure.

Other studies such as the one by Ülger et al. [37] focused on interventions with gifted students in science. Specifically, they developed an intervention based on 3 modules to examine the abilities of gifted students and found positive effects for them, as this level matches their actual educational needs and potential. Another of the included studies based on an enrichment programme is the one developed by García-Perales and Almeida [38], in which the use of technology played a critical role. Among their findings, they found that the 3-week programme based on the implementation of specific educational responses improves children’s adaptation levels and, in some cases, their school performance. In addition, this study raises the question of earlier diagnosis of students with high abilities in order to design educational responses tailored to their needs.

Within the enrichment programme approach, the research conducted by Martín-Lobo et al. [39] may also be found. This focused on an enrichment intervention based on high performance and cognitive skills, creativity and cooperation programmes with 37 primary school students. This led to improvements in attention, creativity and interpersonal problem solving. Another piece of research within the scope of enrichment or extension of the basic curriculum which may be worth mentioning here is presented in the study by Robertson and Pfeiffer [40]. In the American context, this work presents a guide to the application of the RtI model to adjust curricular teaching to the needs of students. One of the potentials of this model is that it is valid for both gifted and standard students, and it contributes to reduce school failure and school segregation. Its results not only lead to improvements in performance, but also to a considerable increase in student motivation by providing cognitive challenges adapted to each student’s abilities.

The studies by Yu and Jen [41] and the one developed by Yoon et al. [42] were included within the enrichment programmes and closely linked to the STEM areas. Concerning the first one, nanotechnology concepts were introduced to 28 high ability students by using a 200-min programme divided into 40-min science sessions twice a week. To achieve this, 4th and 6th grade contents were examined and some activities were specifically designed for them. In all of these, nanotechnology were used by using the 5E instructional model. Results showed that the common curriculum and nanotechnology are perfectly compatible. Students are able to learn these concepts through appropriately-designed activities. Therefore, Yoon et al. [42] focused their study on abilities and skills of 10 gifted and talented students who wanted to become scientists by using the YSTLC programme during a week. They found that intervention improved students’ attitude, STEM knowledge and leadership skills.

The study by Golle et al. [43] was carried out in Germany. This focused on the analysis of the effect of a HCPA enrichment programme in infant education academies by using a quasi-experimental design and in which several knowledge areas were included, apart from the STEM one, so as to satisfy all needs which highly talented students may show. To achieve this, a wide range of elements were included, ranging from those common ones which appear on the curriculum to more transversal areas. More specifically, general cognitive abilities were included, specific mastery skills, specific mastery interests, self-concept and motivation, autorregulation, control and social competences. This programme incremented students’ performance only on Maths and German. However, no increase was measured on any of the other measured variables.

Related to the above, the research of Kahveci et al. [44] proposes going beyond standard enrichment programmes to implement an integrated curriculum model (IMC), this time in the field of social sciences. Under this perspective, it is observed that the combination of teaching content adapted to the cognitive abilities of students with high abilities and the modality of instruction leads to a change in the attitude and perception of students regarding the usefulness of learning in this area.

In contrast to other studies, the study by Doobay et al. [45] was conducted with 41 high-ability students and 41 students with autism. The main purpose of the study was to lay the basis for appropriate early diagnosis between these two groups, which sometimes share certain observable characteristics. In this manner, the cognitive, adaptive and psychosocial functioning of both groups was compared, finding that the high ability students without autism presented a higher processing speed, motor, adaptive and psychosocial functioning skills than the students with autism.

In contrast to most studies that focused on performance, the study by de Oliveira et al. [46] focused on an 8-week social skills training programme with 9 high-ability/gifted children, based on socialisation, communication, expression of feelings, self-management, self-advocacy and assertiveness, and collaboration. After the programme, it was found that both the high ability/gifted children and their teachers and family members noticed improvements in relation to their social behaviour patterns after the implementation of the programme. Finally, the study by Van Der Meulen et al. [47] with Dutch gifted students presents a pullout program, the “Day a Week School” with 25 highly capable students, aimed at reducing socioemotional problems, disruptive behaviour and increasing the self-concept of these students and reducing parental stress. For this purpose, on one day a week gifted students were taken out of their reference class to perform a set of activities with other high-ability students. Following the DWS programme, children showed improved school results, as well as a wide range of positive effects in different areas of social-emotional well-being.

### 3.3. Quality of the Articles Included

**Figure 2:**
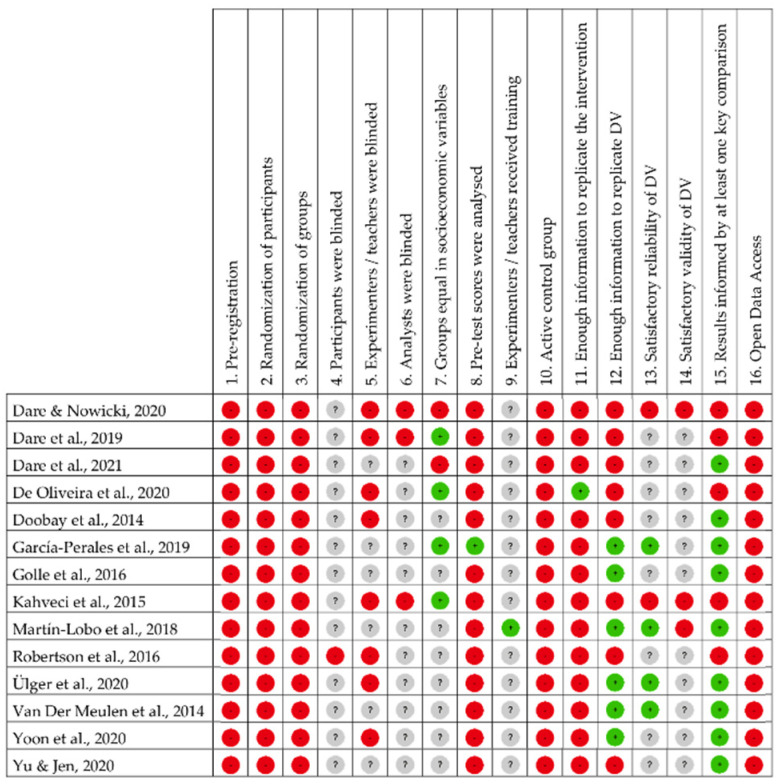
Quality analysis of the articles included.

**Figure 3:**
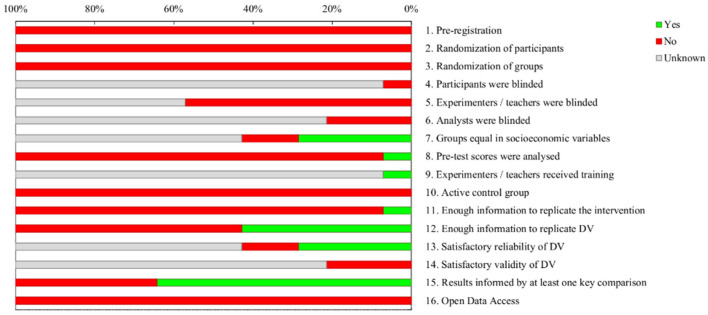
Overview of the quality of the articles included.

Figure 2 shows the detailed findings from the quality analysis of the 14 studies included in the review:

Figure 3 represents the item-by-item quality summary for all studies. From the total number of possible answer alternatives that each paper could reach for each item of the scale, 11.61% reached a positive value, 57.14% a negative value and 31.25% unknown. It should be noted that very few studies answered the quality questions analysed correctly. Sometimes, the positive results come from indirect mentions suggesting fulfilment of the criteria.

Of all the papers analysed, none were pre-registered (item 1) or showed open data (item 16). None conducted any kind of randomised control (items 2 and 3). Notably, none of the studies used an active control group (item 11). In fact, of all the studies reviewed, only two used control group designs. Only one study (7.14%) analysed the dependent variable before the intervention (item 8). In contrast, many of the studies (64.28%) analysed the outcomes after the intervention (item 15). In general terms, it can be stated that most of the studies analysed showed a low methodological quality.

## 4. Discussion

In general terms, the systematic review carried out on 14 articles dealt with different educational interventions with the population with high-ability/high-capacity/gifted students. More specifically, there were two research questions to be answered by this study:

- What kind of educational interventions are being developed with con high abilities/gifted students?

In view of the results obtained, it has been found that there are three modalities of educational interventions carried out with gifted students consistent with previous studies such as the one developed by De Corte [48]. The first one is related to the acceleration of students, considering the supremacy of the positive aspects over the negative ones. The promotion of students to higher grades ensures that the student finds a curriculum adapted to his or her cognitive ability and shares classes with peers of similar maturation to his or her own [36,38]. The second modality is related to keeping students in their class of reference, but implementing curricular enrichment programmes, where the content to be learned goes beyond basic curriculum. These programss have proven to be highly effective for the design of educational responses adapted to the particular needs of the high-ability group [39,40,41,44]. The third one is grouping, which consists of establishing homogeneous classes for gifted learners [47]. De Corte [48] defines pull-out programs as a system in which “high-ability students of a school spend several hours per week in a separate room where they can work under supervision on certain projects of their own choice” (p. 14).

However, based on the findings of the studies included in this review, it is necessary to avoid the application of generalized and standardized programmes to these students, as each one responds to a different profile with very particular needs, hence the importance of designing and implementing integrated curricular programmes adapted to the cognitive abilities of gifted students [39,42]. Another feature to be included in several of the studies within this review is the consideration of psychosocial factors, and in particular, educational and organisational resources specific to each of them as a guarantee to ensure the psycho-emotional balance of students [38,40,46].

As is the case with other studies [2,4,47], these results show that students with high intellectual abilities share the same common characteristics in terms of intelligence and creativity. However, it is a heterogeneous and diverse group to such an extent that each of them have their own characteristics, thus differentiating them from each other. Therefore, it is necessary to develop an appropriate educational response to the specific needs of each student with high abilities, through the design and implementation of specific plans based on the measures and support required.

For this purpose, it may be advisable to carry out an early identification of the characteristics and needs of high ability or gifted students [30,45], and in particular, as stated by Comes, Díaz, Luque and Moliner [4], it may be necessary to carry out a psycho-educational assessment process, understood as:

“a process of collection, analysis and evaluation of relevant information about the different elements involved in the teaching and learning process, in order to identify the educational needs of certain students who present or may present imbalances in their personal and/or academic development, and to base and specify decisions regarding the curricular proposal and the type of help they may need in order to progress in the development of different abilities” (p. 105).

Furthermore, as it has been previously stated in several studies [24,26,49,50,51,52], in order to develop an educational response in response to the needs of each high-ability student and based on a psycho-educational assessment carried out previously, it is essential for teachers to acquire strong training in the educational care of students with high abilities, aimed at promoting educational care whose purpose is to offer students with high abilities educational opportunities to develop their potential and talents to the maximum.

- What perceptions do high-ability/gifted students have about educational measures adopted by schools?

Although the studies reviewed in this systematic review are different, a great convergence has been observed in relation to the analysis of students’ perceptions of the measures adopted by schools. The performance and assessment of students with high abilities will be positive as long as educational measures are adapted to their individual needs and presents them with a cognitive challenge. Otherwise, the likelihood of school failure will increase considerably, either because of boredom or a lack of interest, or due to insufficient psychosocial support resources [32,33,35,40].

These data contrast with the results found in several studies [39,42,53,54] that students with high abilities have a biological and neurological basis in terms of socio-emotional difficulties and, therefore, tend to show low self-esteem and self-perception, as noted in the introduction section [2,5,6].

Thus, to the extent that there is an inadequate adaptation of the teaching-learning process to the characteristics of students with high abilities, this school context, as found in other studies [16,55,56] state, puts these students at a disadvantage, and they tend to become bored, which leads to a general lack of motivation and, therefore, to failure at school.

Therefore, as stated by some authors [16,57,58], it is necessary to design and implement intervention measures appropriate to the needs of each student with high abilities or giftedness in order to favour their own perceptions and attitudes about the measures adopted in response to their particular situation, thus promoting quality education based on inclusion and attention to diversity.

In this sense, general conclusions which may be extracted from this work and concerning data previously shown, it can be confirmed that two intervention modalities exist: first of all, the acceleration concerning an academic year further than the one in which the student has officially joined [35,36,37,38]; secondly, curricular enrichment programmes [38,39,40,41,42]. However, these latter are more disperse and not always fulfill students’ expectations. Nevertheless, concerning the results obtained, if appropriate designs are used, they may better adjust to students’ interests, improve their performance as well as other ways of learning such as social skills, attitude and motivation towards academic purposes or leadership.

This conclusion is undoubtedly linked to the other general conclusion, as it has been detected that students’ perception is not always satisfactory concerning educational attention received in their corresponding schools. This may directly influence their self-esteem levels, as well as learning motivation and performance [34,35].

Due to the fact that this work has been limited to analyzing intervention modalities and students’ perceptions, it would be convenient to advance in further studies when analyzing systems to determine the needs of highly talented students [4]. As was mentioned in the introduction, it can be confirmed that their diagnosis is quite complex to establish attention to diversity measures [1,14,15] and multidimension of learning potential together with social, family or emotional factors [3,16]. This reality makes it necessary to perform further studies on teacher training and cross-professional collaborative work so as to appropriately fulfill these students’ needs.

- What degree of the methodological quality shows the evidence on intervention research for high abilities/gifted students?

The present systematic review has found low quality in relation to interventions delivered to high ability/gifted students. These findings are consistent with the study by Steenbergen-Hu et al. [28] in which they report the low quality of underachievement interventions with gifted students. Previous studies where the quality of educational interventions has been analysed have called for caution in making interpretations based on studies with low methodological quality [32]. In particular, this study states “if all of these results had been collated in a quantitative meta-analysis without a proper analysis of their quality, most likely the conclusions would have been deceivingly positive” [32]. Based on this approach, we believe that generalising the effects found in the included papers could lead to misleading interpretations. Therefore, the results found in this study encourage more research to analyse the effect of educational interventions with higher quality and methodological rigour among this population of pupils. In this regard, studies with pre-post design with an active control group would be the best methodological option when analysing an intervention with the best methodological control. On the other hand, this study has a number of limitations which need to be considered. Firstly, there are those related to the methodological design. The systematic review based on the combination of key words through the use of booleans may limit the search previously performed. Another limitation related to the method is the use of the WOS and Scopus databases, thus leaving aside the grey literature. Nevertheless, this study has a number of strengths that should be considered. This research provides an overview of the educational interventions that have been developed with people with high abilities in the last decade at different educational stages. In this regard, this document may be of great use both for experts within the field of special education and for teachers who have students with these characteristics and need to have a framework of reference to adjust their teaching according to the needs and interests of the students. In view of the results obtained, it is intended that further research should be conducted with experimental and longitudinal designs concerning students with high abilities in order to improve their teaching and learning processes.

## Figures and Tables

**Table 1:** Analysis of selected studies [34,35,36,37,38,39,40,41,42,43,44,45,46,47].

Author	Year	Purpose	Design	Participants	Intervention
Dare et al.	2021	examine how gifted students conceptualized beliefs about grade-based acceleration in inclusive schools	Group concept mapping approach. Mixed method. 2 groups	40	Grade-level acceleration vs. non grade-level acceleration
Dare et al.	2020	Describe the experiences of students who moved into classes with older classmates and explore their attitudes towards grade-based acceleration.	Phenomenological approach. Qualitative. One group.	11	Grade-level acceleration
Ülger et al.	2020	Examine the impact of differentiated inquiry based science lesson modules for gifted students on the students’ scientific process skills (SPS).	Mixed method. Single group pre-test/post-test design	16	Based science lesson modules for gifted students on the students’ scientific process skills (SPS).
De Oliveira et al.	2020	Describe and compare the social skills, behavioral problems, and academic competence of students with High Abilities/Giftedness (HA/G): (1) according to their own account, before and after a program on social skills	Quantitative. Pre-test/post-test design. 2 EG.	9 HA/G, 8 teachers and 8 parents/guardians	THS Social Skills Training Programme
Yu & Jen	2020	Cultivate teachers’ capacity to teach new technology, to enhance K–12 high-ability students’ interests and understanding of basic science, and to produce innovations in future industrial and technological development.	Quantitative. Pre-test/post-test design. One group.	28 high-ability students	Nanotechnology enrichment program
Yoon et al.	2020	Design and explore the effects of an enrichment program on the leadership, attitude, and motivation of ethnic minority gifted and talented students who want to be scientists and engineers in the future	Mixed: Pre-test/post-test, daily reports and interviews. One group.	10 gifted and talented students	Youth Science and Technology Leadership Camp (YSTLC) program.
Dare et al.	2019	Explored 26 high-ability students’ beliefs about important considerations in grade-based acceleration	Mixed mehod. One group.	17	Group concept mapping activities
García-Perales et al.	2019	Analysing the impact of an enrichment programme on children’s adjustment and performance	Quantitative. Pre-test/post-test design: 1 EG and 2 CG	45 (12gifted students and 33 non gifted students)	An enrichment program imparted to a group of students with high intellectual abilities during the academic year 2017/18 over three weekly sessions during school hours, where emerging technologies were an important key in how it was delivered.
Martín-Lobo et al.	2018	This research focus on to provide a project for gifted students with enrichment programs that can be performed at the school level.	Quantitative. Pre-test/post-test design: 6 EG	37	Expanded Curriculum, Creative Literature, Scientific World, Creative Mathematics, Art and Culture and Cooperation programs.
Golle et al.	2016	It presents the results of an HCAP extracurricular enrichment programme in which a basic strategy was implemented in German primary schools.	Quantitative. Pre-test/post-test design: EG and CG	Third-grade students attending the enrichment program (N = 423) and nonattending third-grade students (N = 2328)	HCAP extracurricular enrichment programme
Robertson et al.	2016	This article reports on a study that developed and field tested a procedural guide for implementation of the RtI model with gifted students.	Mixed method. One group.	13 gifted and RtI experts	Response to Intervention (RtI) model in which gifted students are provided with additional curricular material adapted to their level.
Kahveci et al.	2015	Explore individual gifted and talented student views on a differentiated social studies curriculum unit, namely, luckily it is present (good to have it)	Qualitative research methods. One group.	12	Two-month implementation of differentiated social studies instruction
Doobay et al.	2014	Provide an empirical account of the intellectual, adaptive, and psychosocial functioning of high ability youth with and without ASD	Quantitative. Post design. One CG and EG.	82 (41 high abilities and ASD students and 41 high abilities students)	Completed a psychoeducational evaluation to assist with academic planning that included measures of cognitive, academic, and psychosocial functioning
Van Der Meulen et al.	2013	To investigate whether DWS decreases children’s social–emotional and behavior problems and parents’ stress, and improves children’s self-concept, enjoyment at school, and academic achievement	Quantitative. Pre-post design. One group.	89	A pullout program, the ‘‘Day a Week School’’ (DWS)

## Data Availability

The information is available on request to the corresponding author with justifiable reason.

## Supplementary Materials

The following are available online at https://www.mdpi.com/article/10.3390/children8050365/s1, Table S1 PRISMA 2020 Checklist.
